# Off-Label Bone Morphogenetic Protein 2 Use Results in Successful Posterolateral Lumbar Fusion in a Veteran Population

**DOI:** 10.5435/JAAOSGlobal-D-23-00122

**Published:** 2024-10-10

**Authors:** Maria A. Munsch, Jonathan Dalton, Stephen R. Chen, Melissa Tang, Christopher J. Como, James D. Whaley, Shaan D. Sadhwani, Mitchell S. Fourman, Jeremy D. Shaw, Joon Y. Lee

**Affiliations:** From the Department of Orthopaedic Surgery, Division of Spine Surgery, University of Pittsburgh Medical Center, Pittsburgh, PA (Dr. Munsch, Dr. Dalton, Dr. Chen, Dr. Tang, Dr. Como, Dr. Whaley, Dr. Sadhwani, Dr. Fourman, Dr. Shaw, and Dr. Lee); Department of Surgery, Orthopaedic Surgery Service, VA Pittsburgh Medical Center, Pittsburgh, PA (Dr. Munsch, Dr. Dalton, Dr. Chen, Dr. Fourman, Dr. Shaw, and Dr. Lee); the Pittsburgh Orthopaedic Spine Research Group (POSR), Pittsburgh, PA (Dr. Munsch, Dr. Dalton, Dr. Chen, Dr. Tang, Dr. Como, Dr. Whaley, Dr. Shaw, and Dr. Lee); and the Orland Bethel Family Musculoskeletal Research Center (BMRC), Pittsburgh, PA (Dr. Chen, Dr. Tang, Dr. Como, Dr. Shaw, and Dr. Lee)

## Abstract

**Introduction:**

Patients within the US Veterans Health Administration (VA) system have higher rates of comorbidities and chronic pain, increasing risks of complications/poor outcomes following spine surgery. Although the use of bone morphogenetic protein 2 (BMP-2) is established for anterior lumbar interbody fusion, its indications for off-label use in posterolateral fusion are unclear. The objective of this study was to evaluate safety and utility of BMP-2 in posterolateral fusion through a 15-year experience at the VA.

**Methods:**

Patients underwent posterolateral lumbosacral fusions with BMP-2 by a single VA surgeon from January 1, 2005, to January 1, 2020. The primary outcome was fusion assessed through postoperative radiographs. Secondary outcomes included adjacent segment disease (ASD) and postoperative pain clinic utilization.

**Results:**

Sixty-eight patients underwent lumbosacral posterolateral fusion with BMP-2; 77.9% were discharged home and had no postoperative complications. All patients achieved bony fusion at a mean of 113.3 ± 59.9 days postoperatively. Five patients were diagnosed with cancer postoperatively, and eight patients required revision for ASD. No notable predictors of ASD exist. Preoperative opioid use predicted postoperative pain clinic utilization.

**Discussion:**

Posterolateral lumbar fusion with BMP-2 in veterans yields high fusion rates and favorable complication profiles and should be considered in multimorbid hosts.

**Study Design:**

Retrospective review of prospectively collected data.

The demographic and psychosocial makeup of the US veteran population makes treatment of spinal pathology in this group challenging. Patients receiving care through the US Veterans Health Administration (VA) tend to have lower average income and educational level and are more commonly a member of a medically underserved demographic than the general population.^1^ Veteran patients are 14 times more likely to self-report their health status as “poor” when compared with their civilian counterparts.^1^ Smoking is common, with 27% of veterans overall and 43% of those with coronary artery disease reporting active tobacco use.^2^ Severe back pain was reported by 21% of veteran patients, and a markedly higher number of younger veterans reported severe pain (7.8%) compared with civilians.^3^ When compounded with the high prevalence of psychiatric comorbidities such as posttraumatic stress disorder and the extensive prescription of narcotics by VA providers (25% in 2012), this patient population's medical vulnerability necessitates thoughtfully planned elective spine surgery.^4^

Early fusion and pain control are essential when performing posterolateral spinal fusion to achieve symptomatic improvement and return to function. The osteoinductive nature of recombinant human bone morphogenetic protein 2 (BMP-2, INFUSE, Medtronic) promotes cell proliferation, chemotaxis, and differentiation toward osteogenesis.^5,6,7,8^ BMP-2 has been shown to markedly improve the likelihood of fusion among smokers compared with iliac crest bone graft (ICBG) and avoids an additional surgical approach with associated risks of pain, numbness, and infection.^9,10^

However, in comparison to on-label anterior use, the use of BMP-2 in posterolateral fusions is controversial. Classically cited risks of BMP-2 in the spine include seroma, heterotopic bone formation, radiculitis, and theoretical risk of malignancy.^11,12,13,14,15^ Most of these concerns are derived from research of on-label anterior use, and high-quality consensus works largely fail to corroborate these risks.^16,17^

Thorough reports of long-term patient outcomes and complication profiles are necessary to better understand the utility of BMP-2 in a population with numerous comorbidities, specifically in patients undergoing spine surgery at VA facilities. The purpose of this retrospective cohort study is to define the risks and benefits associated with off-label BMP-2 use in posterolateral spinal fusion within a multimorbid veteran patient population. The primary outcome in this study was the achievement of posterolateral fusion. The authors hypothesized that the use of BMP-2 in the veteran population during posterolateral lumbosacral fusions would result in consistent fusion without a markedly increased risk of morbidity or malignancy.

## Methods

Following approval by an institutional review board (Pro00003390), a retrospective analysis of a prospectively collected cohort of consecutive patients who underwent lumbar fusion surgery with BMP-2 augmentation done by a single surgeon (JL) at a Veterans Affairs Hospital from 2005 to 2020 was conducted. This study was deemed exempt from obtaining informed consent under Category 4, subsection ii of the institutional review board approval document. This subsection indicates that information regarding human subjects is recorded by the investigator in such a manner that the identity of the human subjects cannot readily be ascertained and that subjects will not be contacted or reidentified. All included patients underwent a posterolateral fusion surgery for degenerative lumbar stenosis without any anterior column instrumentation. The number of levels fused and whether L5 or S1 were included was recorded. Patients who had previously received BMP-2, sustained acute spinal injuries or required an acute decompression and fusion, or who failed to follow-up for > 1 year were excluded. Large (12-mg vial) kit size of BMP-2 in all cases was morcellized and combined into a mixture, termed a slurry, with unilaterally harvested autogenous ICBG. The accompanying absorbable collagen sponge was divided in half before use. Thorough posterolateral gutter dissection was done in all cases to place the graft slurry following fusion preparation. Indications for ICBG harvesting included revision cases, morbidly obese patients (body mass index > 35), and active smoking status. The ICBG harvest was done by making an incision over the iliac crest, dissecting to bone, and subsequently using a gauge and pituitary to collect bone graft.

Fusion was assessed by both the primary surgeon using radiographs and a chief resident on the spine service. Criteria for assessment of fusion included evidence of contiguous bone on plain radiographs, less than 3 mm of segmental motion on flexion/extension radiographs, and no evidence of radiographic loosening or implant failure.^18,19,20,21^ Regardless of the time line to achievement of fusion, radiographs were obtained at all postoperative clinic visits for each patient. Postoperative clinic visits occurred at intervals of 2 weeks, 6 weeks, 3 months, 6 months, 1 year, and then yearly thereafter.

Patient demographics and comorbidity index, as measured with the Age-Adjusted Charlson Comorbidity Index (ACCI), which has been used previously in spine surgery research, were collected.^22^ Perioperative factors, including surgery characteristics, hospital length of stay, and discharge disposition, were collected. All postoperative complications and outcomes, including infection, new neurologic deficit, venous thromboembolic event, seroma, radiculopathy, heterotopic ossification, and malignancy, were collected. Length of postoperative follow-up, time to fusion, incidence of adjacent segment disease (ASD), and indications for revision surgery were recorded. Preoperative opiate use and postoperative pain clinic utilization were used as indicators of preoperative- and postoperative chronic pain.

All statistical analyses were conducted by the investigators using Prism 9.1 (GraphPad). Univariate analyses to identify predictors of ASD and postoperative pain clinic utilization within this population were conducted using Fisher exact test and Student t-tests where appropriate, with variables with a *P* value of <0.05 on univariate analysis included in a multivariate logistic regression. Variables are cited in text as proportions (percentages) and mean ± standard deviation (median, 95% CI).

## Results

In total, 68 patients (65 males, 95.6%) with an average age of 62.5 ± 10.9 (median 64.5, 95% CI 62.8 to 67.5) years and an average postoperative follow-up time of 3.3 ± 2.2 (median 2.6, 95% CI 1.8-3.0) years were included. Of these, 23 (33.8%) were revision procedures. Mean ACCI was 3.8 ± 1.7 (median 4.0, 95% CI 3.0-5.0), 25/68 (36.8%) were diabetics, and 31/68 (45.6%) were smokers (Table 1). Chronic opioids were prescribed to 32/68 (47.1%) patients, and 13/68 (19.1%) were on an additional long-acting opioid.

**Table 1:** Demographic and Historical Information

Factor	Prevalence (n = 68)
Age	62.5 ± 10.9 (median 64.5, 95% CI 62.8-67.5)
Sex (% Male)	65/68 (95.6%)
BMI	31.2 ± 5.0 (median 30.4, 95% CI 29.5-31.8)
BMI ≥ 40 (%)	4/68 (5.9%)
Smokers (%)	31/68 (45.6%)
Age-adjusted Charlson Comorbidity Index	3.8 ± 1.7 (median 4.0, 95% CI 3.0-5.0)
Preoperative opioid use (%)	32/68 (47.1%)
Preoperative long-acting opioid use (%)	13/68 (19.1%)
Neuropathy (%)	10/68 (14.7%)
Diabetics (%)	25/68 (36.8%)

ACCI = Age-adjusted Charlson Comorbidity Index, BMI = body mass index, CI = confidence interval

**Figure 1:**
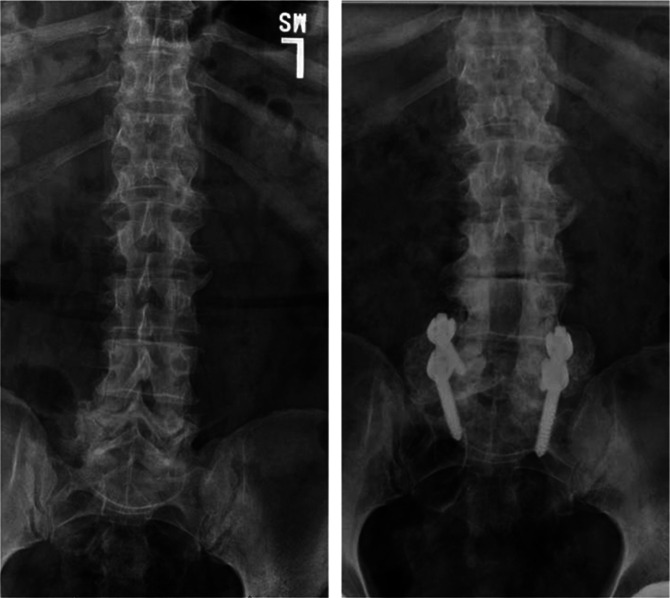
Preoperative and eight-year postoperative radiographs showing bony fusion of a patient who underwent posterolateral fusion with bone morphogenetic protein 2 (BMP-2) augmentation.

A mean 1.8 ± 1.0 levels were fused, and 67 (98.5%) of patients received instrumented fusions. Ten patients (14.7%) underwent ICBG harvesting in addition to the use of BMP-2. Most patients (53/68, 77.9%) were discharged home after a mean hospital length of stay of 4.0 ± 1.5 days. All patients achieved bony fusion at a mean 113.3 ± 59.9 days after surgery, and none developed symptomatic heterotopic ossification (Figure 1). Secondary outcomes are indicated in Table 2. No patients developed a deep infection, and three patients (4.4%) had draining wounds that required an incisional wound vacuum or supplemental oral antibiotics. All drainage resolved without surgical intervention. No seromas formed, although 2 of 68 patients (2.9%) required nonoperative care for a superficial partial wound dehiscence. Of patients with prolonged drainage or superficial dehiscence, none required acquisition of microbial cultures or prolonged antibiotics. The all-cause 90-day readmission rate was 5 of 68 (7.4%), and only one patient had an unplanned return to the OR (1.5%). Five patients (7.3%) received cancer diagnoses at a mean of 2.9 ± 1.8 years after the surgery, of whom only one case (a neuroendocrine tumor of the colon) was diagnosed within 1 year of surgery. Additional malignancies that were diagnosed included colon adenocarcinoma (5.5 years after BMP-2), papillary urothelial carcinoma of the bladder (3.2 years after BMP-2), prostate adenocarcinoma (2.7 years after BMP-2), and a pancreatic neuroendocrine tumor (2.2 years after BMP-2).

**Table 2:** Analysis of Secondary Outcomes

Factor	Prevalence (n = 68)
Iliac crest bone graft used (%)	10/68 (14.7%)
Hospital length of stay (mean)	4.0 ± 1.5 days
Days to bony fusion (mean)	113.3 ± 59.9
All cause 90-day readmission (%)	5/68 (7.4%)
Complications	
Deep infection	0
Draining wounds requiring IVAC/oral Abx (%)	32/68 (47.1%)
Superficial wound dehiscence (%)	2/68 (2.9%)
Unplanned return to OR	1/68 (1.5%)
Cancer diagnosis	5/68 (7.4%)
Years to diagnosis (mean)	2.9 ± 1.8
Colon neuroendocrine tumor (years to diagnosis)	1 (<1)
Colon adenocarcinoma (years to diagnosis)	1 (5.5)
Papillary urothelial bladder carcinoma (years to diagnosis)	1 (3.2)
Pancreatic neuroendocrine tumor (years to diagnosis)	1 (2.2)
Prostate adenocarcinoma (years to diagnosis)	1 (2.7)
Radiographically apparent ASD (%)	16/68 (23.5%)
ASD requiring surgical intervention	8/68 (11.7%)
Years to revision	3.2 ± 2.5 (median 2.2, 95% CI 0.2-7.0)

Abx = antibiotics, ACCI = Age-adjusted Charlson Comorbidity Index, BMI = body mass index, CI = confidence interval, IVAC = incisional wound vacuum, ASD = adjacent segment disease

Radiographically apparent ASD was diagnosed in 16 of 68 patients (23.5%), of whom 8 (11.7%) required surgical intervention. One additional revision surgery was performed for implant failure, which occurred after the initial observation of radiographic fusion in this individual. Revisions occurred a mean of 3.2 ± 2.5 (median 2.2, 95% CI 0.2-7.0) years after the index surgery. No factors were identified as markedly associated with ASD on univariate analysis.

Eighteen patients (26.5%) were followed by a pain clinic at the time of their final postoperative follow-up appointment, representing a 43.8% decrease in chronic pain utilization postoperatively. The only notable predictor of postoperative pain clinic utilization was preoperative opioid use (*P* < 0.001, odds ratio 6.1; Table 3).

**Table 3:** Analysis of Factors Predicting Long-Term Pain Clinic Use Following Posterior Lumbar Fusion With BMP-2 Augmentation

Predictors of Pain Clinic	Pain Clinic (n = 18)	No Pain Clinic (n = 50)	Univariate Analysis (*P*)	Multivariate Logistic Regression (Odds Ratio [95% CI])
Age (years)	58.9 ± 13.6	63.9 ± 9.5	0.095	0.98 (0.9-1.0)
BMI	32.9 ± 3.9	30.6 ± 5.3	0.1	
Smoking (%)	11/18 (61.1%)	20/50 (40.0%)	0.17	
Age-adjusted Charlson Comorbidity Index	3.2 ± 2.0	4.0 ± 1.5	0.1	
Preoperative opioid use (%)	15/18 (83.3%)	17/50 (34.0%)	0.001^a^	**6.1 (1.7-26.9)**
Neuropathy (%)	1/18 (5.6%)	9/50 (18.0%)	0.3	
Baseline ambulation with cane/walker (%)	9/18 (50.0%)	15/50 (30.0%)	0.2	
No. of levels fused	2.2 ± 1.0	1.6 ± 0.9	0.03^a^	1.4 (0.8-2.7)
Revision procedure (%)	9/18 (50.0%)	14/50 (28.0%)	0.1	
Fusion to L5 (%)	11/18 (61.1%)	34/50 (68.0%)	0.8	
Fusion to S1 (%)	6/18 (33.3%)	14/50 (28.0%)	0.8	
Revision after BMP-2 (%)	5/18 (27.8%)	4/50 (8.0%)	0.05^a^	3.8 (0.7-21.9)
Adjacent segment disease (%)	6/18 (33.3%)	10/50 (20.0%)	0.3	

ASD = adjacent segment disease, ACCI = Age-adjusted Charlson Comorbidity Index, BMI = body mass index, BMP-2 = bonemorphogenetic morphogenic protein 2, CI = confidence interval

a Indicates significance of *P* values < 0.05.

Bold represents the factor that found to be predictor of postoperative pain clinic use.

## Discussion

The successful management of spinal pathology in vulnerable and comorbid hosts such as those in the VA healthcare system remains a challenge. BMP-2 was first studied in spine surgery as an adjunct to anterior lumbar interbody fusions to be used as an alternative to ICBG.^23^ BMP2 was FDA approved in single-level ALIFs based on favorable initial findings. However, many of these works were industry sponsored,^11,24^ and subsequent studies have reported adverse effects such as excessive bone growth, seroma/hematoma formation, arachnoiditis, retrograde ejaculation, infections, and cancer.^12,13,14,15,24^ It is difficult to extrapolate the utility of BMP2 in anterior fusion to posterolateral fusion. These two regions of the spine have vastly different vascular beds available in the prepared intervertebral space compared with the transverse processes.^25^ In addition, these two fusion techniques are vastly different from a biomechanical standpoint—ALIF involves a compressive process facilitating fusion, whereas posterolateral fusion represents a distractive process.^25^ To the authors' knowledge, BMP-2 augmentation of posterolateral lumbar fusions in degenerative conditions has not been sufficiently addressed in vulnerable populations such as patients undergoing spine surgery at the VA.

In this study, all patients underwent posterolateral lumbar fusions augmented with BMP-2. All patients achieved the primary outcome of successful posterolateral fusion. No wound infections were found. Wound drainage that resolved without return to the operating room was reported in 4.4% of cases, and only one patient (1.5%) required an unplanned return to the OR. This patient likely sustained a postoperative inflammatory seroma and had an optimal result after débridement. The 100% fusion rate and other excellent perioperative outcomes noted in this study are similar to prior work on BMP-2, but it is notable that these results are achievable in vulnerable hosts and with a high percentage of successful fusions in cases done for revision procedures.^26^ Among the VA patients undergoing surgery in this study, nearly half were smokers, nearly half were on opioids preoperatively, more than one third were diabetic patients, and the average BMI was in the obese range, with nearly 6% having a BMI of more than 40. Although prospective comparative trials are required to broadly extrapolate these claims, this work generally supports the use of BMP-2 in multimorbid hosts.

A meta-analysis of all works comparing BMP-2 and ICBG by Wu et al^27^ reported higher fusion rates, better patient-reported outcomes, lower blood loss, fewer subsequent surgical procedures, and shorter surgical times with BMP-2. Although the findings of Wu et al and other meta-analyses are supportive of BMP-2, Galimberti et al^28^ noted that heterogeneity in study quality and the preparation, dosing, and use of BMP-2 may impede the reliability of pooled analyses. The present work is unique in that its patient population is relatively homogenous with high rates of smoking, systemic comorbidities, and chronic pain.^1,2,3,4^ Our findings of high rates of fusion, few complications, and a drop in pain clinic utilization of more than 40% are supportive of the use of BMP-2 in vulnerable hosts.

Five patients were diagnosed with cancer over the course of their follow-up period, but there are several confounding factors that make drawing a causal link between BMP-2 exposure and malignancy difficult in this study. One possible confounding factor includes the relatively long average length of time between surgery and the cancer diagnoses (2.9 ± 1.8 years). In addition, the average age of diagnosis of cancer among our patients mirrors the age of diagnosis reported in a recent national study of cancer among VA patients.^29^ That same national study also reported that the incidence of cancer among VA patients was similar to civilian averages, but that VA patients were diagnosed at an earlier stage.^29^ This earlier stage of cancer diagnosis among VA patients may be another confounding factor in our analysis because it may represent improved screening within a cohesive healthcare system such as the VA, which is the largest integrated provider of cancer care in the United States.^29^ In addition, patients in this study have substantial cancer risks including medical comorbidities and lifestyle factors, such as smoking and poor diet, that are markedly more common among VA patients.^29^ Finally, it is difficult to use cancer type to infer a possible effect of BMP-2 because the most common three types of cancer noted in the national VA population (prostate, lung/bronchus, and colon/rectum) are also the three most commonly studied types of cancer in patients treated with BMP-2.^29,30^ Of the five cancer diagnoses in this study, only a pancreatic neuroendocrine tumor is outside the top four most cited tumors in the national VA population.^29^ Notably, this cancer diagnosis was also the only one made within 1 year of spine surgery, but it is difficult to draw more definitive conclusions regarding the causal relationship of BMP-2 on this particular malignancy.

Prior investigations on the effect of BMP-2 use in spine surgery on cancer risk have produced mixed results. A recent large systematic review by Skovrlj et al^30^ found no studies that demonstrated BMP-2 use causing cancer de novo but did find 43% of included studies, suggesting that BMP-2 enhances tumor function. In addition, Carragee et al^11^ reported a markedly increased risk of cancer in their randomized controlled trial of BMP-2 in posterolateral arthrodesis (OR at 2 years 3.37, 95% CI 1.89-5.56). However, a methodological issue in their analysis was the authors' inclusion of multiple episodes of localized basal and squamous cell carcinomas of the skin from the same patients in their study, which is not consistent with the reportable malignancy criteria set forth by the Commission on Cancer.^31^ Kelly et al^32^ in their analysis of 467,916 Medicare patients found that not only BMP-2 was not associated with an increased risk of cancer but also a mild protective effect was identified in women (OR 0.93, 95% CI 0.9-0.97). Importantly, few prior works include a mean follow-up of more than 5 years, and there is not currently consensus on how long patients ought to be monitored to assess cancer risk of osteobiologics. It is also difficult to attribute causality for cancers that occur well after surgery.

Eight patients (11.7%) required surgical intervention for symptomatic ASD at an average of 3.2 ± 2.5 years after their index surgery, which is consistent with previously reported incidence of surgically treated ASD of 13.6% at 5 years in posterior fusions and more than 30% at 8 to 10 years for all fusion types.^33,34^ There is notable disagreement on the risk factors for ASD. A meta-analysis of variable quality identified age, BMI, smoking status, hypertension, preoperative adjacent disk disease, long segment fusions, preoperative superior facet violation, and sagittal imbalance as risk factors for ASD.^35^ However, more homogeneous cohort studies identified fewer risk factors and found only preoperative degeneration at the adjacent level as a risk factor for ASD in their cohort with a minimum 5-year follow-up.^36^ As prior biomechanical studies have noted increased adjacent level stresses with more rigid fusion constructs, there is concern that the robust fusion masses typical of BMP-2–mediated fusions may increase the risk of ASD.^37^ Although no work to our knowledge has evaluated this risk, ASD and junctional failure remain risks of lumbar fusion, and therefore, spine surgeons should monitor for these sequelae in BMP-2 recipients.^33,38-40^

A notable proportion (47.1%) of patients in this work used chronic short-acting and/or long-acting opioids preoperatively. Preoperative opioid use was the only notable predictor of postoperative pain clinic utilization in this work, and pain clinic utilization decreased by more than 40% postoperatively. This trend in opioid utilization postoperatively reflects that reported by prior works.^41^ In this review, the lack of a comparison group does not permit the determination of whether there is a relative benefit of the use of BMP-2 in chronic pain patients. An evaluation of patients undergoing lumbar fusions who used one or more preoperative opioid medications using the Multi-Payer Claims Database failed to identify a benefit of BMP-2 in a predominately younger and female patient population.^42^ However, pain medication utilization is dependent on system, physician, and host and therefore is likely to differ regionally and by health system. Changes in prescriber protocols and the use of an opioid dashboard tool in the electronic health record were implemented in 2013 as part of the Veterans Administration Opioid Safety Initiative, thereby distinguishing it from civilian prescriber programs.^43^ Future higher-quality studies to evaluate the benefit of BMP-2 on chronic pain utilization within a specifically VA population are therefore necessary.

Beyond the limitations inherent to retrospective analyses, this work has several limitations. Its most prominent limitation is the relatively small numbers, which were likely responsible for our confidence intervals, which cross 1.0, and the lack of a comparison group. Because of the relatively wide confidence intervals, these results, although encouraging, lack power and must be interpreted with caution. Without a control group, this work must be classified as a case series rather than a prospective study. Both of these factors somewhat limit the substantive analyses possible in this work. Although we had the option to include a comorbidity-matched cohort of civilian patients operated on by the same surgeon without BMP-2, we did not believe that doing so would be a fair comparison due to the differing logistics, prescriber patterns, and host demographics between institutions. Our analysis also did not include female patients. This is the result of the male skew in the veteran population, not patient selection. Regarding radiographic assessment of fusion, this was done through postoperative radiograph, rather than CT, due to cost constraints at VA and was done by the operative surgeon, rather than a blinded evaluator. In addition, the use of ICBG as indicated to supplement the fusion mixture can be considered a confounding factor. Finally, this work does not include patient-reported outcomes, and instead relies on chronic pain utilization to assess surgical benefit. Future prospective works should feature randomization, blinded unbiased radiographic evaluation, patient-reported outcomes, and mid-term (>5 year) follow-up.

## Conclusion

Within the limitations of this study, off-label use of BMP-2 as an adjunct for elective posterolateral spinal fusions in this veteran population appears to result in a high rate of fusion, low postoperative pain clinic utilization, few complications, and an acceptable incidence of ASD. Further work is needed to more extensively validate the efficacy, safety, and benefit of the use of BMP-2 among comorbid populations, such as the veteran population examined in this study.
